# Global Validation of a Process-Based Model on Vegetation Gross Primary Production Using Eddy Covariance Observations

**DOI:** 10.1371/journal.pone.0110407

**Published:** 2014-11-06

**Authors:** Dan Liu, Wenwen Cai, Jiangzhou Xia, Wenjie Dong, Guangsheng Zhou, Yang Chen, Haicheng Zhang, Wenping Yuan

**Affiliations:** 1 State Key Laboratory of Earth Surface Processes and Resource Ecology, Beijing Normal University, Beijing, China; 2 Chinese Academy of Sciences, Institute of Botany, State Key Laboratory of Vegetation and Environmental Change, Beijing, China; 3 Chinese Academy of Meteorological Sciences, Beijing, China; Ecole Pratique des Hautes Etudes, France

## Abstract

Gross Primary Production (GPP) is the largest flux in the global carbon cycle. However, large uncertainties in current global estimations persist. In this study, we examined the performance of a process-based model (Integrated BIosphere Simulator, IBIS) at 62 eddy covariance sites around the world. Our results indicated that the IBIS model explained 60% of the observed variation in daily GPP at all validation sites. Comparison with a satellite-based vegetation model (Eddy Covariance-Light Use Efficiency, EC-LUE) revealed that the IBIS simulations yielded comparable GPP results as the EC-LUE model. Global mean GPP estimated by the IBIS model was 107.50±1.37 Pg C year^−1^ (mean value ± standard deviation) across the vegetated area for the period 2000–2006, consistent with the results of the EC-LUE model (109.39±1.48 Pg C year^−1^). To evaluate the uncertainty introduced by the parameter V*_cmax_*, which represents the maximum photosynthetic capacity, we inversed V*_cmax_* using Markov Chain-Monte Carlo (MCMC) procedures. Using the inversed V*_cmax_* values, the simulated global GPP increased by 16.5 Pg C year^−1^, indicating that IBIS model is sensitive to V*_cmax_*, and large uncertainty exists in model parameterization.

## Introduction

Terrestrial gross primary production (GPP) is the largest carbon flux in terrestrial ecosystems, and it is approximately 20 times larger than the amount of carbon introduced from anthropogenic sources [1]. Thus, even small fluctuations in GPP can cause large changes in the airborne fraction of carbon and subsequently influence future climate change [2]. Vegetation also contributes to human welfare by providing food, fiber and energy [3]–[4]. Therefore, regular monitoring and reliable estimation of global terrestrial GPP is important for improving our understanding of the global carbon cycle, accurately predicting future climate, and ensuring the long-term sustainability of terrestrial ecosystem services.

Ecosystem models serve as a backbone for evaluating large-scale and global GPP. Two categories of ecosystem models are widely used: process-based and satellite-based. Satellite-based models are driven by remotely sensed data and provide simple means of estimating GPP [5]; however, they are limited in their ability to model mechanisms. Process-based models typically exhibit detailed expressions of terrestrial processes, such as photosynthesis, respiration, phenology, and hydrological cycle. Therefore, process-based models play important roles in investigating the mechanisms underlying current biases in estimated ecosystem production [6], predicting the future conditions of the terrestrial carbon cycle, and exploring its feedback to climate change [7].

Numerous attempts have been made to develop and improve process-based models. However, a recent study from the North American Carbon Project (NACP) showed that current models perform poorly and difference between observations and simulations far exceed the observational uncertainty [8]. Model parameter uncertainty is a key source limiting the accuracy of process-based models. Knorr and Heimann analyzed the uncertainties of process-based models [9]. They found that parameter uncertainties could explain much of the large variance among models and that the largest uncertainties arose from plant photosynthesis, respiration and soil water storage.

The maximum rate of carboxylation by the enzyme Rubisco (V*_cmax_*) is fundamental in modeling photosynthesis [10]. Sensitivity analysis shows that the projections of ecosystem production are particularly sensitive to the fixed parameters associated with V*_cmax_* [11]. Therefore, the parameterization scheme of V*_cmax_* is essential for GPP simulation, and its impacts on model performance need to be tested.

Eddy covariance measurements recorded by the increasing number of eddy covariance (EC) towers provide a great opportunity for model validation and improvement. Concurrent measurements include carbon fluxes, latent heat, and sensible heat, as well as meteorological conditions such as air temperature and relative humidity, and provide unprecedented datasets for model validation and evaluations of parameter constraints [12]. The current network of EC sites covers a wide range of ecosystem types, thus it has the potential to significantly improve our understanding of the variation in GPP across time, space and biomes [13].

The goal of this study was to validate a process-based ecosystem model (Integrated BIosphere Simulator, IBIS) based on measurements from 62 EC sites [14]. The specific objectives were to (1) examine the performance of IBIS over several ecosystem types, (2) compare IBIS model performance with a satellite-based model (i.e., Eddy Covariance-Light Use Efficiency, EC-LUE), and (3) investigate the impacts of the parameter V*_cmax_* on model performance.

## Data and Methods

### 2.1 IBIS model and parameter inversion

The Integrated BIosphere Simulator (IBIS) is designed to integrate a variety of terrestrial ecosystem processes within a single, physically consistent modeling framework. It represents land surface processes, canopy physiology, vegetation phenology, long-term vegetation dynamics, and carbon and water cycling [14]. The photosynthesis module of the IBIS model is provided by the formulations of Farquhar [15].

C_3_ photosynthesis and C_4_ photosynthesis are expressed separately in the IBIS model. For C_3_ plants, the gross photosynthesis rate per unit leaf area, 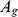
(mol CO_2_ m^−2^ s^−1^) is expressed as
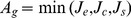
(1)where 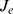 is light-limited rate of photosynthesis, 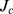 represents the Rubisco-limited rate of photosynthesis, and 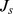 is the photosynthesis limited by the inadequate rate of utilization of triose phosphate.

The light-limited rate of photosynthesis is given as
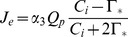
(2)where 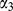 is the intrinsic quantum efficiency of CO_2_ uptake in C_3_ plants (mol CO_2_ mol^−1^ quanta), 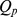 is the flux density of photosynthetically active radiation absorbed by leaf (mol quanta m^−2^ s^−1^), 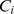 is the concentration of CO_2_ in the intercellular air spaces of the leaf (mol mol^−1^), and 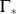 is the compensation point for gross photosynthesis (mol mol^−1^).

The Rubisco-limited rate of photosynthesis is calculated as
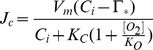
(3)where 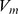 is the maximum carboxylase capacity of Rubisco (mol CO_2_ m^−2^ s^−1^) and 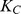 and 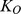 are the Michaelis-Menten coefficients (mol mol^−1^) for CO_2_ and O_2_, respectively.

Under conditions of high intercellular CO_2_ concentrations and high irradiance, photosynthesis is limited by the inadequate rate of utilization of triose phosphate. This limitation is expressed as
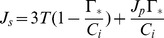
(4)where 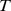 is the rate of triose phosphate utilization.

Photosynthesis in C_4_ plants is similarly modeled as the minimum of three potential capacities to fix carbon [16]. The gross photosynthesis rate is given by
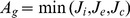
(5)where 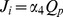 is the light-limited rate of photosynthesis, 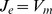 is the Rubisco-limited rate of photosynthesis and 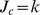 is the CO_2_-limited rate of photosynthesis at low CO_2_ concentrations.

The parameter V*_cmax_* (mol CO_2_ m^−2^ s^−1^) is very important for simulating the photosynthesis process. In the IBIS model, it is established as a constant that differs among plant functional types (PFTs) (Table 1). To validate the IBIS model and investigate the impact of the V*_cmax_* parameter scheme on model performance, we conducted two simulations (IBIS and IBIS-Type) for each site. IBIS simulation used the default values of V*_cmax_* (Table 1). For the IBIS-Type simulation, the vegetation-specific V*_cmax_* values were inversed for each PFT. The Markov Chain-Monte Carlo (MCMC) procedure was used for the parameter inversion, and the Metropolis-Hastings (M-H) algorithm was used as the MCMC sampler [17]–[18] (see Xu et al. [19] and Yuan et al. [20] for detailed descriptions of the MCMC procedure). We conducted 10000 samples for each site and assigned the V*_cmax_* value with the highest frequency as the optimal value of V*_cmax_*. Finally, we ran the model using the inversed V*_cmax_* for each PFT as the IBIS-Type simulation.

**Table 1:** The values of V*_cmax_* set in the IBIS model and the initial range of MCMC inversion.

Plant Functional Type (PFT)	V*_cmax_* (10^−6^ mol CO_2_ m^−2^ s^−1^)	V*_cmax_* range (10^−6^ mol CO_2_ m^−2^ s^−1^)
Tropical broadleaf trees	65	1–300
Warm-temperate broadleaf trees	40	1–300
Temperate broadleaf trees	30	1–300
Boreal broadleaf trees	30	1–300
Temperate conifer trees	30	1–300
Boreal conifer trees	20	1–300
Shrub	27.5	1–300
C3 herbaceous	25	1–300
C4 herbaceous	15	1–300

V*_cmax_* set according to Kucharik et al. [47].

### 2.2 EC-LUE model

A satellite-based model (i.e. EC-LUE) [5], [21]–[23] was used to compare the local and global GPP simulations with those of the IBIS model. The EC-LUE model is based on two assumptions: (1) ecosystem GPP has a direct relationship with the absorbed photosynthetically active radiation (APAR) via light use efficiency (LUE), where LUE is defined as the amount of carbon produced per unit of APAR; and (2) realized LUE may be reduced below its theoretical potential value by environmental stressors, such as low temperatures or water shortages. The EC-LUE model is driven by four variables: the normalized difference vegetation index (NDVI), photosynthetically active radiation (PAR), air temperature, and the ratio of sensible to latent heat flux (Bowen ratio).

### 2.3 Data

We used the eddy covariance (EC) data to validate the IBIS model. We used data obtained from the LaThuile dataset (http://www.fluxdata.org). The daily GPP values were estimated from the eddy covariance measurements using a community standard method [24]. Briefly, GPP was estimated from the equation:
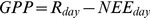
(6)where 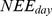 is daytime NEE. Daytime ecosystem respiration 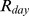 was estimated using daytime temperature and an equation describing the temperature dependence of respiration, which was formulated from nighttime NEE measurements. For further details on the algorithm, see Reichstein et al. (2005) [24]. The gap filling and quality control of this dataset are conducted according to standard criteria [25]–[26], and the uncertainty in annual GPP can be controlled to some extent below 100 g C m^−2^ year^−1^ [25]. This dataset is widely used in model studies. In the present study, we selected 62 EC sites for model validation (Table S1). The selected data covered six major terrestrial biomes: evergreen needleleaf forest (ENF), deciduous broadleaf forest (DBF), evergreen broadleaf forest (EBF), mixed forest (MF), grassland (GRA) and savanna (SAV). Additional information on the vegetation, climate and soil characteristics of each site was collected from the associated metadata of the LaThuile dataset. Daily average, maximum and minimum temperature, relative humidity, precipitation, cloud fraction, photosynthetically active radiation, latent heat and sensible heat were used to drive the IBIS, and GPP was used to evaluate its performance.

To decrease model uncertainty, we used the satellite-based leaf area index (LAI) from the Moderate Resolution Imaging Spectroradiometer (MODIS) as a model input. The Normalized Difference Vegetation Index (NDVI) data derived from MODIS were used to drive the EC-LUE model. The 8-day MODIS-NDVI data (MOD13) and the MODIS-LAI (MOD15) data with 1-km spatial resolution were used for model verification at the EC sites. Quality control (QC) flags, which signal cloud contamination in each pixel, were examined to filter out NDVI and LAI data of insufficient quality. We temporally filled missing or unreliable values for each 1-km MODIS pixel based on their corresponding quality assessment data fields, as proposed by Zhao et al. [27]. In addition, data on soil properties, including soil texture, organic carbon content and nitrogen content, were required to input soil information into the model, and were therefore collected from the sites where EC towers are established.

For global simulation, we used meteorological datasets from the Modern Era Retrospective Analysis for Research and Applications (MERRA) archive for 2000–2006 to drive the IBIS and EC-LUE models. MERRA is a NASA reanalysis dataset for the satellite era which uses a new version of the Goddard Earth Observing System Data Assimilation System Version 5 (GEO-5). We used climate conditions at 10 meters above the land surface and at a resolution of 0.5° latitude by 0.6° longitude. The Global Gridded Surfaces of Selected Soil Characteristics datasets were used to supply soil properties for the IBIS model; detailed information is available from the website (http://ww.isric.org). The global distribution of plant functional types (PFTs) was derived by overlapping the MODIS land-cover type product with the Köppen-Geiger climate classification map, with the land cover classifications aggregated into nine PFTs (Table 1). The IBIS model was unable to simulate carbon cycle processes in cropland or wetland; therefore, these two vegetation types were replaced with C_3_ grassland in this study.

### 2.4 Statistical analysis

Three metrics were used to evaluate model performance:

(1) the coefficient of determination, R^2^, which represents how much variation in the observations is explained by the model simulations;

(2) the root mean square error (RMSE), which represents the total difference between the simulated and measured values;

(3) the relative predictive error (RPE), which represents the ratio of error to observation. It is computed as
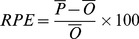
(7)where 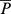 and 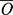 are the mean simulated and measured values, respectively.

One-way ANOVA was employed using SPSS software to test the significance of the differences in optimal V*_cmax_* for each biome in the IBIS-Type scheme, and the paired-samples t-test was used to test the significance of the difference in statistical metrics between different models.

## Results

### 3.1 Model validation at EC towers

**Figure 1:**
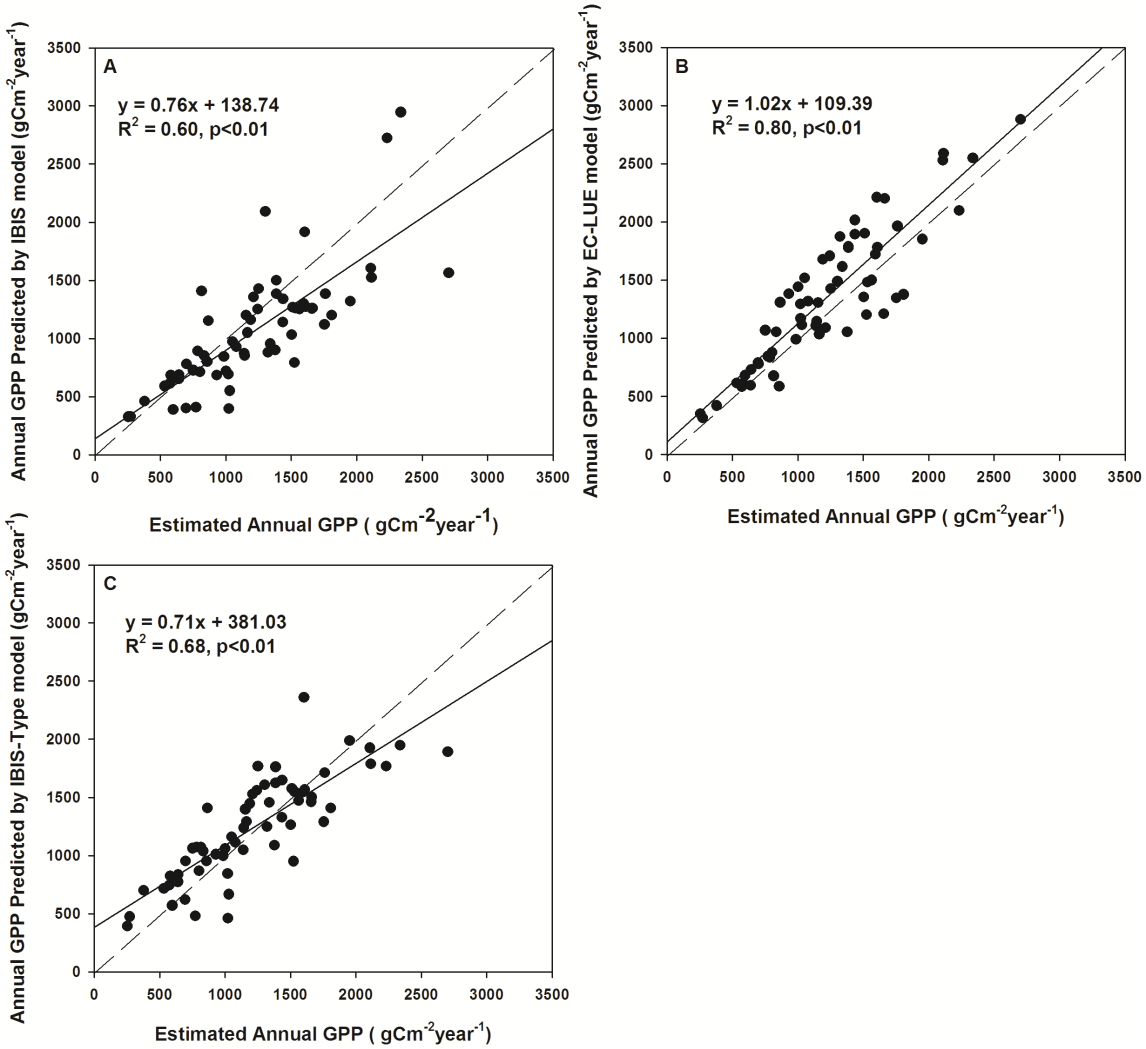
Comparison of predicted and measured GPP. Comparison between gross primary production (GPP) estimated from Eddy Covariance (EC) measurements and GPP predicted from different model simulations: (a) IBIS, (b) EC-LUE and (c) IBIS-Type. The solid lines are the linear regression lines and the short dashed lines are the 1∶1 lines.

**Figure 2:**
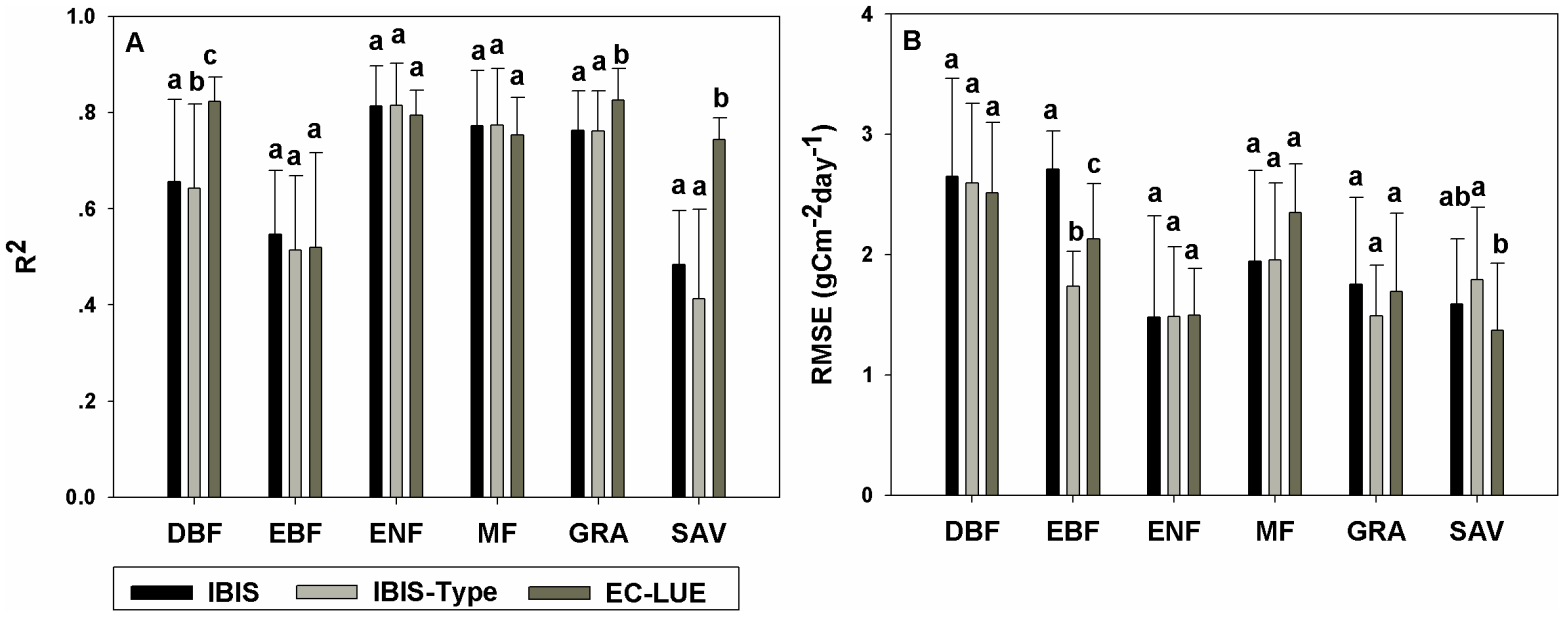
Comparison among models for different PFTs. Comparisons among IBIS, IBIS-Type and EC-LUE models for each plant functional type (PFT), where (a) and (b) are the results of R^2^ and RMSE, respectively. Lowercase letters above the bars indicate significant differences among models.

The overall comparison of the estimated GPP with the EC measurements showed that the IBIS model performed well in capturing the variability in GPP. Across all study sites, the IBIS model explained approximately 60% of the variation in site-averaged GPP (Fig. 1). The coefficient of determination (R^2^) varied from 0.11 at the ES-LMa site to 0.94 at the CA-Man site, with a mean value of 0.71 across all EC sites. The mean R^2^ values were 0.66, 0.55, 0.81, 0.77, 0.76 and 0.48 for deciduous broadleaf forest, evergreen broadleaf forest, evergreen needleleaf forest, mixed forest, grassland and savanna, respectively (Fig. 2). The root mean square error (RMSE) varied from 1.48 to 2.71 g C m^−2^ day^−1^ among the six vegetation types (Fig. 2). On average, the mean relative predictive error (RPE) of all sites was −9.68%, and the RPE at most sites was less than 20% (Table 2).

**Table 2:** The performance of the IBIS model and the EC-LUE model.

Site	Vegetation Type	IBIS			EC-LUE		
		R^2^	RMSE	RPE	R^2^	RMSE	RPE
CA-Oas	DBF	0.75	2.57	−32.34	0.87	2.26	26.75
DE-Hai	DBF	0.84	2.26	−17.92	0.88	1.62	−3.42
FR-Fon	DBF	0.68	2.98	−24.83	0.77	3.23	22.55
FR-Hes	DBF	0.74	3.07	−20.36	0.80	2.72	9.49
IT-Col	DBF	0.75	2.62	−18.94	0.75	2.88	20.36
IT-Ro1	DBF	0.61	2.03	6.49	0.77	2.33	32.39
IT-Ro2	DBF	0.24	4.05	−25.93	0.83	2.85	30.68
US-Bar	DBF	0.80	1.80	1.10	0.83	2.82	37.93
US-Bn2	DBF	0.72	1.12	10.49	0.86	1.04	14.97
US-Dk2	DBF	0.68	3.74	−23.88	0.81	2.86	19.87
US-Ha1	DBF	0.76	2.82	−19.57	0.82	2.38	11.84
US-MOz	DBF	0.32	3.62	−24.46	0.85	3.02	34.81
US-UMB	DBF	0.64	2.57	−2.44	0.92	2.47	41.20
US-Wi8	DBF	0.65	1.85	19.52	0.76	2.72	37.90
AU-Tum	EBF	0.69	2.80	22.60	0.75	2.27	−5.86
AU-Wac	EBF	0.43	2.92	24.27	0.39	2.66	5.82
FR-Pue	EBF	0.63	2.89	59.41	0.62	2.03	15.37
PT-Mi1	EBF	0.44	2.24	72.49	0.33	1.55	−17.23
CA-Ca3	ENF	0.74	1.38	14.26	0.76	1.87	14.11
CA-Man	ENF	0.94	0.61	6.83	0.80	1.19	13.54
CA-Obs	ENF	0.91	0.80	−11.11	0.86	1.16	9.49
CA-Ojp	ENF	0.88	0.77	17.75	0.78	0.99	5.13
CA-Qfo	ENF	0.84	0.91	4.25	0.80	1.19	9.92
CA-SJ3	ENF	0.79	1.24	33.48	0.75	2.12	51.35
FI-Hyy	ENF	0.87	1.43	−22.85	0.89	1.20	−2.87
FI-Sod	ENF	0.85	0.89	2.78	0.77	1.06	−7.73
IT-Ren	ENF	0.73	1.49	−7.29	0.69	1.74	−12.11
NL-Loo	ENF	0.81	2.28	−32.99	0.78	1.95	−21.84
RU-Fyo	ENF	0.87	2.30	−34.64	0.85	1.82	−23.40
RU-Zot	ENF	0.86	1.05	−14.51	0.85	1.00	0.63
SE-Fla	ENF	0.86	0.98	13.78	0.76	1.46	6.35
US-Bn1	ENF	0.75	0.99	6.62	0.76	1.10	1.76
US-Fmf	ENF	0.73	1.09	−14.31	0.73	1.50	21.17
US-Ha2	ENF	0.87	1.61	−21.33	0.85	1.96	11.45
US-Ho1	ENF	0.92	2.11	−31.29	0.84	1.69	−9.90
US-Me2	ENF	0.61	2.73	−47.52	0.77	1.63	−20.61
US-Me3	ENF	0.75	0.89	−6.74	0.76	1.15	−31.66
US-NC2	ENF	0.71	4.05	−42.25	0.83	2.08	6.32
CA-Let	GRA	0.84	1.68	−34.70	0.90	1.10	13.64
CN-HaM	GRA	0.82	1.68	−42.69	0.83	1.47	12.36
CN-Xi2	GRA	0.67	0.77	21.88	0.70	0.82	10.69
DE-Meh	GRA	0.84	1.90	−25.53	0.85	1.49	0.20
HU-Bug	GRA	0.70	1.92	−30.21	0.85	2.30	46.43
IE-Dri	GRA	0.65	2.80	−33.36	0.75	2.25	−6.00
IT-MBo	GRA	0.84	2.32	−28.74	0.83	2.49	20.46
PT-Mi2	GRA	0.76	1.43	−24.61	0.77	2.14	52.77
RU-Ha1	GRA	0.79	0.86	−3.50	0.82	1.77	42.51
US-ARc	GRA	0.64	2.91	−33.45	0.90	2.21	41.67
US-Aud	GRA	0.84	1.03	16.20	0.89	0.57	10.06
BE-Bra	MF	0.81	1.31	12.90	0.71	1.91	0.95
BE-Jal	MF	0.90	1.48	−19.78	0.84	1.99	−4.06
BE-Vie	MF	0.82	1.88	−25.86	0.78	2.10	−27.43
CA-Gro	MF	0.75	1.72	−7.45	0.75	3.07	44.54
JP-Tef	MF	0.54	2.29	0.07	0.61	2.33	9.86
JP-Tom	MF	0.78	3.48	−34.21	0.83	2.55	−21.88
US-PFa	MF	0.81	1.44	−0.10	0.76	2.50	28.45
ES-LMa	SAV	0.59	1.55	−39.30	0.77	1.27	8.16
ZA-Kru	SAV	0.32	2.10	−51.83	0.74	1.35	22.69
BW-Ma1	SAV	0.42	1.58	−48.15	0.74	0.90	9.73
US-FR2	SAV	0.11	2.17	−46.41	0.77	1.27	8.16
US-Ton	SAV	0.50	1.48	−20.37	0.79	2.14	31.26
US-SRM	SAV	0.59	1.49	4.82	0.66	1.88	25.23

DBF: deciduous broadleaf forest; EBF: evergreen broadleaf forest; ENF: evergreen needleleaf forest; GRA: grassland; MF: mixed forest; SAV: savanna.

Although the IBIS model explained most of the GPP variability at individual sites, large differences between the predicted and estimated GPP values from the EC measurements were apparent at some sites and for some vegetation types. The IBIS model underestimated GPP for the majority of PFTs and overestimated GPP in evergreen broadleaf forest. Specifically, over 40 of the 62 sites had negative RPE values, and the RPEs of 26 sites were below −20%. The largest underestimation occurred at two savanna sites, ZA-Kru and BW-Ma1, with PREs of −51.83% and −48.15%, respectively. The other 24 sites with RPEs below −20% were predominantly deciduous broadleaf forest (6 sites), evergreen needleleaf forest (7 sites) and grassland (8 sites). In addition, the model overestimated GPP at 6 sites with RPEs greater than 20%, four of which were evergreen broadleaf forest. Extreme overestimation occurred at two sites of evergreen broadleaf forest, FR-Pue and PT-Mi1, with RPEs of 59.41% and 72.49%, respectively.

### 3.2 Comparison of IBIS and EC-LUE

Compared to the satellite-based EC-LUE model, the IBIS model performed comparably at most sites, according to the R^2^, RMSE and RPE values (Table 2). The IBIS mean R^2^ values for evergreen broadleaf forest, evergreen needleleaf forest, mixed forest and grassland were similar to those of the EC-LUE model, which were 0.52, 0.79, 0.75, 0.83, respectively; no significant differences were found for most PFTs. Comparable results were also found for the RMSE, and no significant differences in RMSE were detected for any PFTs except for evergreen broadleaf forest.

However, the EC-LUE model had some advantages for some PFTs (Figure 2). For broadleaf forest, grassland and savanna, the R^2^ of the EC-LUE was significantly higher than that of the IBIS, particularly for savanna, where the mean was 76% higher than that of the IBIS (0.74 for EC-LUE and 0.42 for IBIS). In addition, the EC-LUE had significantly low model error for evergreen broadleaf forest, with a mean RMSE 22% lower than that of the IBIS (2.12 g C m^−2^ day^−1^ for EC-LUE and 2.71 g C m^−2^ day^−1^ for IBIS).

### 3.3 IBIS Performance with inversed V*_cmax_*

Model parameterization did not significantly improve the performance of the IBIS, as indicated by the R^2^ and RMSE. The mean R^2^ values of the IBIS-Type were 0.64, 0.51, 0.81, 0.77, 0.76, 0.41 for deciduous broadleaf forest, evergreen broadleaf forest, evergreen needleleaf forest, mixed forest, grassland and savanna, respectively. These values were very similar to those of the IBIS model; only the R^2^ of the deciduous broadleaf forest differed significantly, being higher in the IBIS-Type model. The overall R^2^ increased from 0.60 to 0.68 after revising V*_cmax_* (Fig. 1). The RMSEs of the IBIS and the IBIS-Type models were also comparable. The mean RMSE of the IBIS-Type varied from 1.49 g C m^−2^ day^−1^ for evergreen needleleaf forest to 2.60 g C m^−2^ day^−1^ for deciduous broadleaf forest. Evergreen broadleaf forest was the only vegetation type yielding a significant difference in RMSE between the two models.

**Figure 3:**
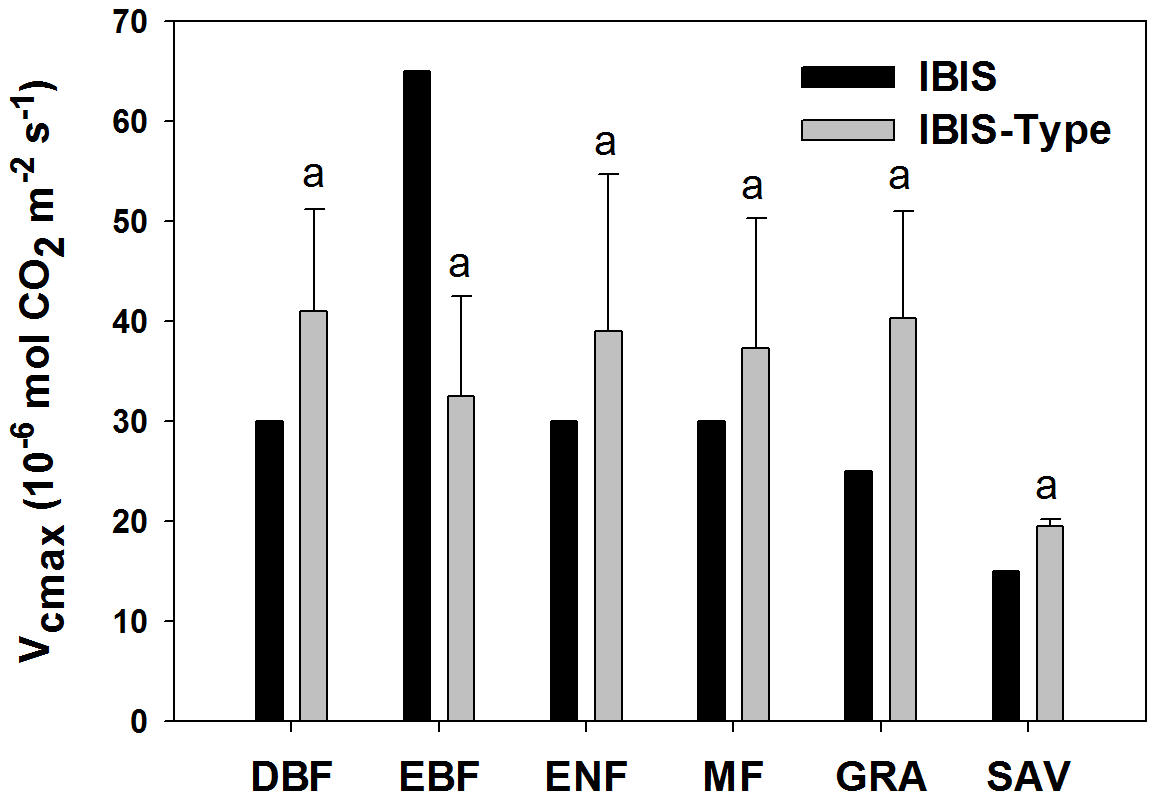
Comparison of V*_cmax_* from the original scheme and the inversed V*_cmax_* values. Comparison of model V*_cmax_* values with the inversed values of the IBIS-Type. Same letter on the top of the bar indicates no significant difference among biomes.

The IBIS employs a set of parameter values for a given PFT (Table 1). In the IBIS-Type scheme, the V*_cmax_* value, which was set to as the mean value of V*_cmax_* inversed from each site within a given PFT, was largely differentiated from the original parameter values. V*_cmax_* of the IBIS-Type was 36.67%, 30.00%, 24.29%, 61.09% and 30.00% higher than that of the IBIS for deciduous broadleaf forest, evergreen needleleaf forest, mixed forest, grassland and savanna, respectively; it was 50% lower for evergreen broadleaf forest. In addition, the V*_cmax_* value inversed at each site did not indicate significant differences among different PFTs (Fig. 3).

### 3.4 Temporal and spatial patterns in global averaged GPP

**Figure 4:**
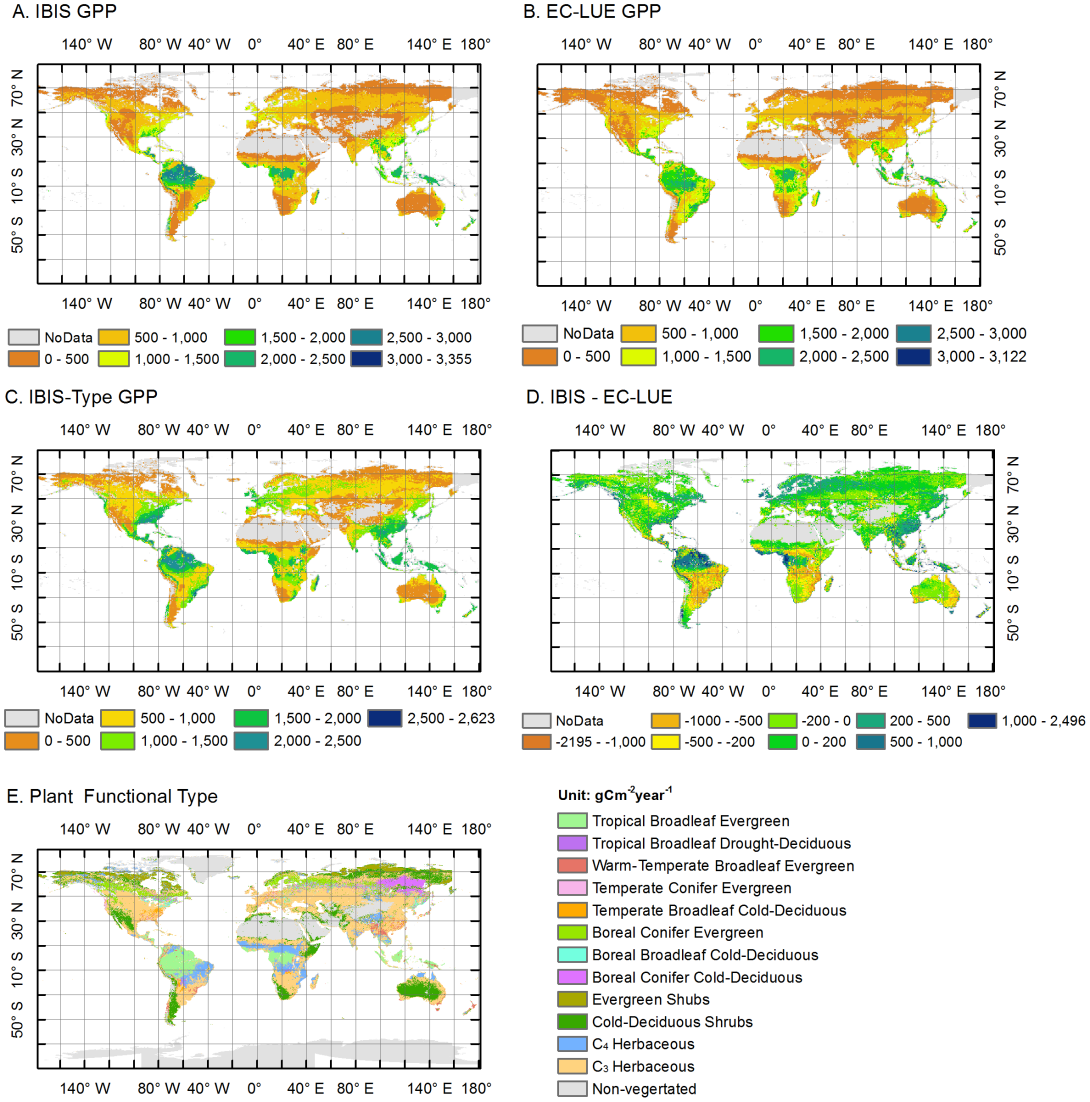
The global pattern of GPP. The global pattern of annual vegetation gross primary production (GPP) from 2000 to 2006. (a) estimated GPP using the IBIS model, (b) estimated GPP using the EC-LUE model, (c) estimated GPP using the IBIS-Type model, (d) the difference between the IBIS and EC-LUE models and (e) the spatial distribution of plant functional types (PFTs).

The spatial pattern in average annual GPP estimated using the original IBIS, the IBIS-Type and the EC-LUE models from 2000 to 2006 were generally consistent (Fig. 4). The highest value was from the humid tropics (Amazonia, Central Africa and Southeast Asia), with an annual GPP over 2000 g C m^−2^ year^−1^. Temperate regions had intermediate levels of GPP, and the lowest GPP was found in both cold and arid regions.

**Figure 5:**
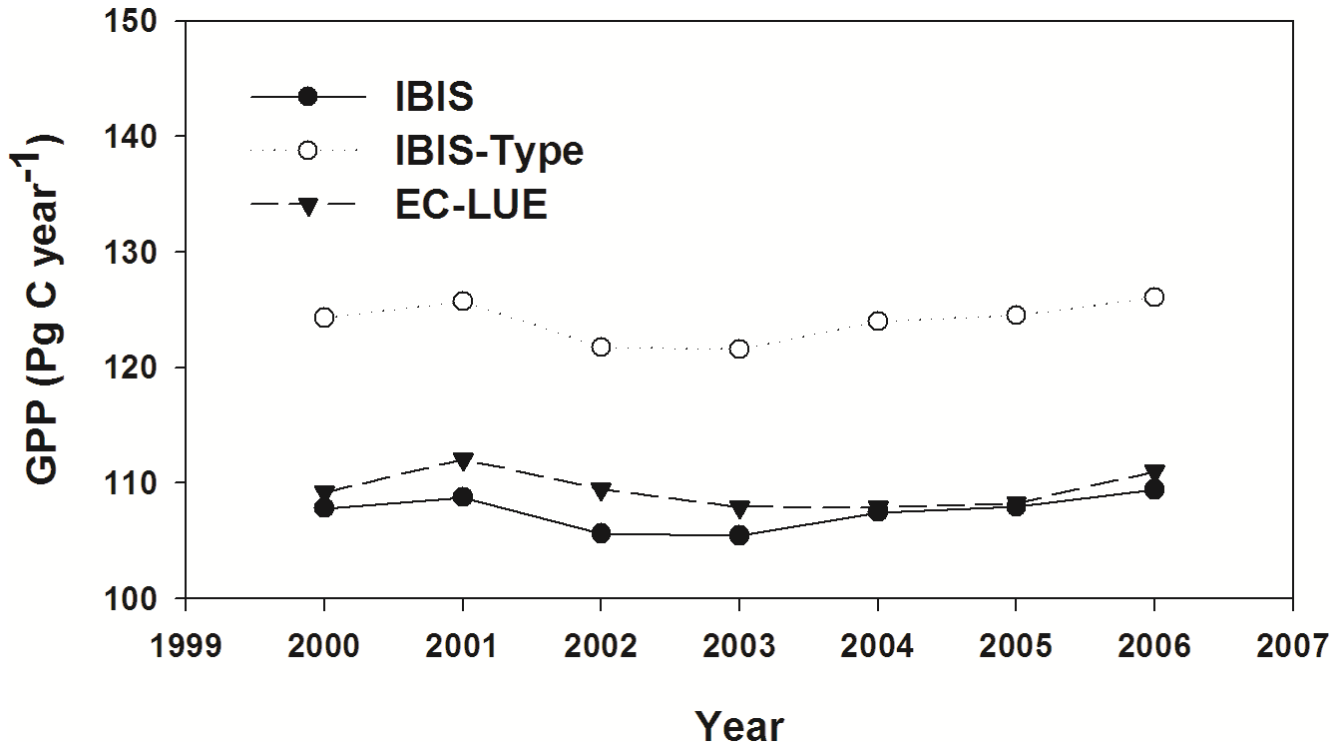
Interannual variability in GPP derived from different models. Interannual variability in global mean gross primary production (GPP) derived from the IBIS, IBIS-Type and EC-LUE models.

The magnitude of GPP estimated by the IBIS and EC-LUE models were comparable, reaching 107.50±1.37 Pg C year^−1^ and 109.39±1.48 Pg C year^−1^ (mean value ± standard deviation) globally, respectively (Fig. 5). Two-model comparisons revealed consistent GPP estimations for the various PFTs (Table 3) with the exception of savanna, for which the IBIS model greatly underestimated GPP. The GPP estimate of the IBIS-Type scheme was much higher than those of the other two models, with a global value of 123.97±1.76 Pg C year^−1^ (Fig. 5). Larger GPP estimations using the IBIS-Type simulation were found for most biomes (Table 3).

**Table 3:** The magnitude of gross primary production (GPP) in each plant functional type (PFT).

GPP (kgCm^−2^year^−1^)	DBF	EBF	ENF	MF	GRA	SAV
IBIS	0.89	2.00	1.22	1.19	0.68	0.63
IBIS-TYPE	1.05	1.68	1.51	1.53	0.89	0.74
EC-LUE	0.78	1.76	0.96	1.16	0.73	0.92

## Discussion

Process-based ecosystem models are one of the most important components of earth system models used to predict future climate change [1]. The IPCC AR5 requested that all earth system models integrate the global carbon cycle module [7]. Previous studies have shown that the uncertainty in carbon cycle models can produce 40% differences in the predicted temperature by 2100 [28]. The GPP is the total photosynthetic uptake or carbon assimilation by plants, and it is a key component of terrestrial carbon balance. Any errors in the GPP simulations will propagate through the model, introducing errors into the simulated biomass and net ecosystem fluxes. If the simulated GPP is too low or too high, predicted leaf area index, wood biomass, crop yield, and soil biomass may also be too low or too high [29].

In this study, we examined the performance of the IBIS, which has been widely used to evaluate the regional and global terrestrial ecosystem carbon balance and has been integrated into earth system models (e.g., Brazilian Earth System) [30]. Our results indicate that the IBIS model is a good candidate for simulating GPP at regional-to-global scales, and its performance was comparable to that of the satellite-based EC-LUE model based on EC site validation and comparison. The magnitude of global GPP estimated by the IBIS is also consistent with results of previous studies. Our estimate of annual global mean GPP was 107.50±1.37 Pg C year^−1^(mean value ± standard deviation). Beer et al. estimated global GPP as 123.8 Pg C year^−1^ [31]. Two satellite-based light-use efficiency models revealed similar estimates of global GPP: 109.29 Pg C year^−1^ by the MODIS algorithm [27] and 111 Pg C year^−1^ by the EC-LUE [21]. Interestingly, we found that the IBIS was consistent with the EC-LUE model across different PFTs (Table 3), despite the large differences between the two approaches (i.e., satellite-based vs. process-based).

However, the IBIS did not perform well for two PFTs: evergreen broadleaf forest and savanna. Among the four evergreen broadleaf forest sites, two (PT-Mi1 and FR-Pue) have subtropical Mediterranean climate with dry summers and wet winters [32]–[33], and the AU-Tum site also suffers drought in summer [34]. At these sites, rainfall is the key driver of water and carbon fluxes [35]. Leuning analyzed the CO_2_ and H_2_O fluxes at the AU-Tum site [34], and found that carbon uptake was more strongly constrained by water stress than by temperature, and strongly affected by soil water availability. Moreover, savannas are characterized by climate with distinct wet and dry seasons, and this climate forcing causes savanna to form open, heterogeneous woodland canopies with grass understories [36].

**Figure 6:**
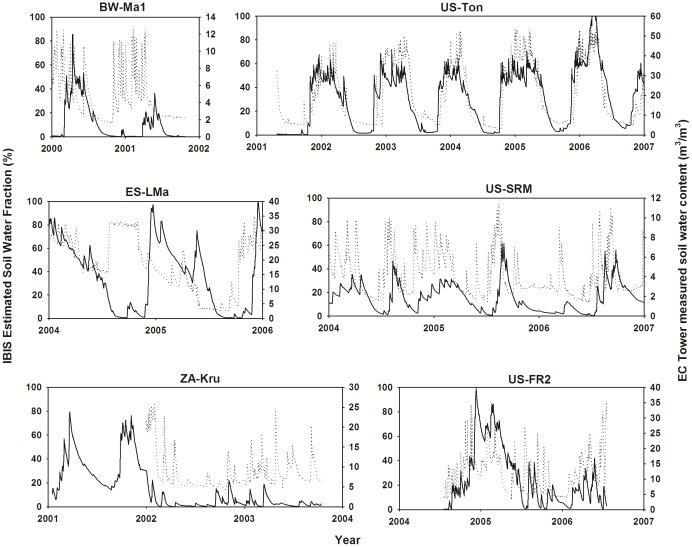
Comparison of estimated and observed soil water. Daily variation in the estimated soil water fraction of the IBIS model (i.e., fraction of soil pore space containing liquid water) and in the observed soil water content at EC sites. The solid lines represent simulation data, and the dotted lines represent the observed data.

These particular ecosystem properties result in greater complexity of modeling fluxes [37]. Process-based models need to simulate variation in soil moisture and plant phenology. However, previous studies have identified significant biases when simulating soil moisture [38]. We examined the performance of the IBIS on soil water at the savanna sites and also found obvious differences between the simulated values and the EC measurements (Fig. 6). Moreover, the IBIS integrated temperature-dominated phenology algorithms developed by Botta et al. [39]. However, field studies suggest that for many drought-deciduous species, the first large precipitation event at the start of the rainy season initiates rapid leaf flush [40]–[41], and leaf senescence is closely related to soil water availability in the dry season [41]–[42]. This relationship may explain why the IBIS model did not effectively capture the variance in GPP at savanna sites.

Parameterization is another large source of uncertainty in process-based models. V*_cmax_* is the key parameter of the photosynthesis process [11], and a study by Bonan et al. [43] suggests that uncertainty in this parameter could account for 30 Pg C year^−1^ variations in model estimation of global GPP. In the present study, the differences in setting V*_cmax_* values between the IBIS and IBIS-Type schemes caused an increase in global GPP of over 16.5 Pg C year^−1^, which also indicated that V*_cmax_* is a salient parameter for simulating GPP. Unfortunately, the determination of V*_cmax_* in current models contains large uncertainty. Rogers surveyed V*_cmax_* in current state-of-the-art models [44] and found that V*_cmax_* varied within a wide range of −46 to +77% of the PFT mean. Thus, the determination of V*_cmax_* and the reduction of uncertainty in this parameter are important issues for model development.

Many parameters in process-based models are established by PFTs, which are based on the assumption that the same type of vegetation responds similarly to the environment. However, a current study found that model parameters were more variable than previously assumed within the given PFTs [45] and that categorization of vegetation into less than eight PFTs may result in artificial multiple steady-states in a model of the Earth's climate-vegetation system depending on the number of PFTs used [46]. In the present study, the variation analysis showed that V*_cmax_* did not significantly differ among PFTs. The predetermined parameterization scheme that sets the V*_cmax_* constant values for each PFT in the IBIS model may cause systematic error.

We attempted to test the impact of V*_cmax_* on model performance. However, variations in V*_cmax_* cannot explain the overall uncertainty of the IBIS model. Validation of the IBIS on three flux sites demonstrated that parameterization and formulations of phenology also limit the model's ability to capture seasonal fluctuations in carbon and water exchange [38]. Particularly in regions with summer drought, phenology is primarily controlled by water supply rather than temperature [34]. This relationship hinders model simulation because biases in phenology and the dynamics of the leaf area index can affect the simulation of evapotranspiration. Such errors can pass to simulations of soil water content and other variables associated with the water and carbon cycles [38]. Therefore, revisions of not only the parameterization of V*_cmax_* but also other parameters and formulations are needed for model improvement.

## Summary

Process-based models are important tools for carbon cycle research, but current models incorporate substantial uncertainty. This study examines the performance of the IBIS model at global EC sites. Our results showed that the IBIS model explained 60% of the variation in GPP at all EC sites and performed comparably to the EC-LUE model, which explained 80% of the variation in observed GPP. At the global scale, the magnitudes of GPP estimated by the IBIS and EC-LUE models were comparable, being 107.50±1.37 Pg C year^−1^ and 109.39±1.48 Pg C year^−1^, respectively. The parameter V*_cmax_* is a key parameter in the photosynthesis model. In the IBIS model, V*_cmax_* was set as a constant for each PFT. The inversed V*_cmax_* value was largely differentiated from the original setting, and no significant differences were detected among PFTs.

## Supporting Information

Table S1 Information of EC sites used in this study.
(DOCX)
