# Supplementary material for: Global Validation of a Process-Based Model on Vegetation Gross Primary Production Using Eddy Covariance Observations
Source: PLoS One. 2014 Nov 6;9(11):e110407. doi: 10.1371/journal.pone.0110407 (PMC4222824; doi:10.1371/journal.pone.0110407)
Supplement: Table S1 — Information of EC sites used in this study. (DOCX) [file pone.0110407.s001.docx]

Table S1. Information of EC sites used in this study

| Site | Latitude, Longitude | Vegetation type | Available years | References |
| --- | --- | --- | --- | --- |
| CA-Oas | 53.63°N, 106.20°W | DBF | 2000-2005 | Griffis et al. (2003) |
| DE-Hai | 51.08°N, 10.45°E | DBF | 2000-2006 | Knohl et al. (2003) |
| FR-Fon | 48.48°N, 2.78°E | DBF | 2005-2006 | / |
| FR-Hes | 48.67°N, 7.06°E | DBF | 2000-2006 | Granier et al. (2000) |
| IT-Col | 41.85°N, 13.59°E | DBF | 2004-2006 | Valentini et al. (1996) |
| IT-Ro1 | 42.41°N, 11.93°E | DBF | 2000-2006 | Rey et al. (2002) |
| IT-Ro2 | 42.39°N, 11.92°E | DBF | 2002-2006 | Tedeschi et al. (2006) |
| US-Bar | 44.06°N, 71.29°W | DBF | 2004-2005 | Jenkins et al. (2007) |
| US-Bn2 | 63.92°N, 145.38°W | DBF | 2003 | / |
| US-Dk2 | 35.97°N, 79.10°W | DBF | 2003-2005 | Liu et al. (2005) |
| US-Ha1 | 42.54°N, 72.17°W | DBF | 2000-2006 | Urbanski et al. (2007) |
| US-MOz | 38.74°N, 92.20°W | DBF | 2004-2006 | Gu et al. (2006) |
| US-UMB | 45.56°N, 84.71°W | DBF | 2000-2003 | Curtis et al. (2002) |
| US-Wi8 | 46.72°N, 91.25°W | DBF | 2002 | Noormets et al. (2007) |
| AU-Tum | 35.66°S, 148.15°E | EBF | 2001-2006 | van-Gorsel et al. (2007) |
| AU-Wac | 37.43°S, 145.19°E | EBF | 2005-2007 | / |
| FR-Pue | 43.74°N, 3.60°E | EBF | 2000-2006 | Rambal et al. (2004) |
| PT-Mi1 | 38.54°N, 8.00°W | EBF | 2003-2005 | David et al. (2004) |
| CA-Ca3 | 49.53°N, 124.90°W | ENF | 2001-2004 | Humphreys et al. (2006) |
| CA-Man | 55.88°N, 98.48°W | ENF | 2000-2003 | Griffis et al. (2003) |
| CA-Obs | 53.99°N, 105.12°W | ENF | 2000-2005 | Bergeron et al. (2007) |
| CA-Ojp | 53.92°N, 104.69°W | ENF | 2000-2005 | Griffis et al. (2003) |
| CA-Qfo | 49.69°N, 74.34°W | ENF | 2003-2006 | Bergeron et al. (2007) |
| CA-SJ3 | 53.88°N, 104.65°W | ENF | 2004-2005 | Amiro et al. (2006) |
| FI-Hyy | 61.85°N, 24.29°E | ENF | 2000-2006 | Rannik et al. (2002) |
| FI-Sod | 67.36°N, 26.64°E | ENF | 2001-2004, 2006 | Rinne et al. (2000) |
| IT-Ren | 46.59°N, 11.43°E | ENF | 2000-2006 | Marcolla et al. (2005) |
| NL-Loo | 52.17°N, 5.74°E | ENF | 2000-2006 | Dolman et al. (2002) |
| RU-Fyo | 56.46°N, 32.92°E | ENF | 2002-2006 | / |
| RU-Zot | 60.80°N, 89.35°E | ENF | 2002-2004 | Wirth et al. (2002) |
| SE-Fla | 64.11°N, 19.46°E | ENF | 2001-2002 | Wallin et al. (2001) |
| US-Bn1 | 63.92°N, 145.38°W | ENF | 2003 | Liu et al. (2005) |
| US-Fmf | 35.14°N, 111.73°W | ENF | 2005-2006 | Dore et al. (2008) |
| US-Ha2 | 42.54°N, 72.18°W | ENF | 2004 | Hadley et al. (2002) |
| US-Ho1 | 45.20°N, 68.74°W | ENF | 2000-2004 | Falge et al. (2002) |
| US-Me2 | 44.45°N, 121.56°W | ENF | 2003-2005 | Thomas et al. (2009) |
| US-Me3 | 44.32°N, 121.61°W | ENF | 2004-2005 | Vickers et al. (2009) |
| US-NC2 | 35.80°N, 76.67°W | ENF | 2005-2006 | / |
| CA-Let | 49.71°N, 112.94°W | GRA | 2000-2005 | Flanagan et al. (2002) |
| CN-HaM | 37.37°N, 101.18°E | GRA | 2002-2003 | Kato et al. (2004) |
| CN-Xi2 | 43.55°N, 116.67°E | GRA | 2006 | / |
| DE-Meh | 51.28°N, 10.66°E | GRA | 2004-2006 | / |
| HU-Bug | 46.69°N, 19.60°E | GRA | 2003-2006 | Gilmanov et al. (2007) |
| IE-Dri | 51.99°N, 8.75°W | GRA | 2003-2005 | / |
| IT-MBo | 46.02°N, 11.05°E | GRA | 2003-2006 | Gilmanov et al. (2007) |
| PT-Mi2 | 38.48°N, 8.02°W | GRA | 2004-2006 | Gilmanov et al. (2007) |
| RU-Ha1 | 54.73°N, 90.00°E | GRA | 2002-2004 | Marchesini et al. (2007) |
| US-Arc | 35.55°N, 98.04°W | GRA | 2005-2006 | / |
| US-Aud | 31.59°N, 110.51°W | GRA | 2005-2006 | / |
| BE-Bra | 51.31°N, 4.52°E | MF | 2000-2002, 2004-2006 | Carrara et al. (2003) |
| BE-Jal | 50.56°N, 6.07°E | MF | 2005 | / |
| BE-Vie | 50.31°N, 6.00°E | MF | 2000-2006 | Aubinet et al. (2001) |
| CA-Gro | 48.22°N, 82.16°W | MF | 2003-2004 | McCaughey et al. (2006) |
| JP-Tef | 45.06°N, 142.11°E | MF | 2001-2002, 2004-0005 | / |
| JP-Tom | 42.74°N, 141.51°E | MF | 2001-2003 | Hirano et al. (2003) |
| US-PFa | 45.95°N, 90.27°W | MF | 2000,2003 | Davis et al. (2003) |
| ES-LMa | 39.94°N, 5.77°W | SAV | 2004-2005 | / |
| ZA-Kru | 25.02°S, 31.50°E | SAV | 2001-2003 | Scholes et al. (2001) |
| BW-Ma1 | 19.92°S, 23.56°E | SAV | 2000-2001 | Arneth et al. (2006) |
| US-FR2 | 29.95°N, 98.00°W | SAV | 2004-2006 | Heinsch et al. (2004) |
| US-Ton | 38.43°N, 120.97°W | SAV | 2001-2006 | Ma et al. (2007) |
| US-SRM | 31.82°N, 110.87°W | SAV | 2004-2006 | Scott et al. (2009) |

DBF: deciduous broadleaf forests; EBF: evergreen broadleaf forest; ENF: evergreen needleleaf forests; GRA: grassland; MF: mixed forests; SAV: savannas.

**References**

Arneth A, Veenendaal EM, Best C, Timmermans W, Kolle O, Montagnani L, Shibistova O (2006) Water use strategies and ecosystem-atmosphere exchange of CO_2_ in two highly seasonal environments. Biogeosciences 3: 421-437.

Aubinet M, Chermanne B, Vandenhaute M, Longdoz B, Yernaux M, Laitat E (2001) Long term carbon dioxide exchange above a mixed forest in the Belgian Ardennes. Agricultural and Forest Meteorology 108: 293-315.

Amiro BD, Barr AG, Black TA, Iwashita H, Kljun N, McCaughey JH, Morgenstern K, Murayama S, Nesic Z, Orchansky AL, Saigusa N (2006) Carbon, energy and water fluxes at mature and disturbed forest sites, Saskatchewan, Canada. Agricultural and Forest Meteorology 136(3-4): 237-251.

Bergeron O, Margolis HA, Black TA, Coursolle C, Dunn AL, Barr AG, Wofsy SC (2007) Comparison of CO_2_ fluxes over three boreal black spruce forests in Canada. Global Change Biology 13: 89-107.

Curtis PS, Hanson PJ, Bolstad P, Barford C, Randolph JC, Schmid HP, Wilson KB (2002) Biometric and eddy-covariance based estimates of annual carbon storage in five eastern North American deciduous forests. Agricultural and Forest Meteorology 113(1-4): 3-19.

Carrara A, Kowalski AS, Neirynck J, Janssens IA, Yuste JC, Ceulemans R (2003) Net ecosystem CO_2_ exchange of mixed forest in Belgium over 5 years. Agricultural and Forest Meteorology 119(3-4): 209-227.

David TS, Ferreira MI, Cohen S, Pereira JS, David JS (2004) Constraints on transpiration from an evergreen oak tree in southern Portugal. Agricultural and Forest Meteorology 122(3–4): 193-205.

Davis KJ, Bakwin PS, Yi CX, Berger BW, Zhao CL, Teclaw RM, Isebrands JG (2003) The annual cycles of CO_2_ and H_2_O exchange over a northern mixed forest as observed from a very tall tower. Global Change Biology 9(9): 1278-1293.

Dolman AJ, Moors EJ, Elbers JA (2002) The carbon uptake of a mid-latitude pine forest growing on sandy soil. Agricultural and Forest Meteorology 111(3): 157-170.

Dore S, Kolb TE, Montes-Helu M, Sullivan BW, Winslow WD, Hart SC, Kaye JP, Koch GW, Hungate BA (2008) Long-term impact of a stand replacing fire on ecosystem CO_2_ exchange of a ponderosa pine forest. Global Change Biology 14(8): 1801-1820.

Falge E, Baldocchi D, Tenhunen J, Aubinet M, Bakwin P, Berbigier P, Bernhofer C, Burba G, Clement R, Davis KJ, Elbers JA, Goldstein AH, Grelle A, Granier A, Guomundsson J, Hollinger D, Kowalski AS, Katul G, Law BE, Malhi Y, Meyers T, Monson RK, Munger JW, Oechel W, Paw KT, Pilegaard K, Rannik U, Rebmann C, Suyker A, Valentini R, Wilson K, Wofsy S (2002) Seasonality of ecosystem respiration and gross primary production as derived from FLUXNET measurements. Agricultural and Forest Meteorology 113(1-4): 53-74.

Flanagan LB, Wever LA, Carlson PJ (2002) Seasonal and interannual variation in carbon dioxide exchange and carbon balance in a northern temperate grassland. Global Change Biology 8(7): 599–615.

Griffis TJ, Black TA, Morgenstern K, Barr AG, Nesic Z, Drewitt GB, Gaumont-Guay D, McCaughey JH (2003) Ecophysiological controls on the carbon balances of three southern boreal forests. Agricultural and Forest Meteorology 117(1-2): 53-71.

Granier A, Ceschia E, Damesin C, Dufrêne E, Epron D, Gross P, Lebaube S, Le Dantec V, Le Goff N, Lemoine D, Lucot E, Ottorini JM, Pontailler JY, Saugier B (2000) The carbon balance of a young Beech forest. Functional Ecology 14(3): 312–325.

Gu LH, Meyers T, Pallardy SG, Hanson PJ, Yang B, Heuer M, Hosman KP, Riggs JS, Sluss D, Wullschleger SD (2006) Direct and indirect effects of atmospheric conditions and soil moisture on surface energy partitioning revealed by a prolonged drought at a temperate forest site. Journal of Geophysical Research 111(D16).

Gilmanov TG, Soussana JF, Aires L, Allard V, Ammann C, Balzarolo M, Barcza Z, Bernhofer C, Campbell CL, Cernusca A, Cescatti A, Clifton-Brown J, Dirks OM, Dore S, Eugster W, Fuhrer J, Gimeno C, Gruenwald T, Haszpra L, Hensen A, Ibrom A, Jacobs AFG, Jones MB, Lanigan G, Laurila T, Lohila A, Manca G, Marcolla B, Nagy Z, Pilegaard K, Pinter K, Pio C, Raschi A, Rogiers N, Sanz MJ, Stefani P, Sutton M, Tuba Z, Valentini R, Williams ML, Wohlfahrt G (2007) Partitioning European grassland net ecosystem CO_2_ exchange into gross primary productivity and ecosystem respiration using light response function analysis. Agriculture Ecosystems and Environment 121(1-2): 93-120.

Humphreys ER, Black TA, Morgenstern K, Cai T, Drewitt GB, Nesic Z, Trofymow JA (2006) Carbon dioxide fluxes in coastal Douglas-fir stands at different stages of development after clearcut harvesting. Agricultural and Forest Meteorology 140(1-4): 6-22.

Hadley JL, Schedlbauer JL (2002) Carbon exchange of an old-growth eastern hemlock (Tsuga canadensis) forest in central New England. Tree Physiology 22(15-16): 1079-1092.

Hirano T, Hirata R, Fujinuma Y, Saigusa N, Yamamoto S, Harazono Y, Takada M, Inukai K, Inoue G (2003) CO_2_ and water vapor exchange of a larch forest in northern Japan. Tellus Series B-Chemical and Physical Meteorology 55(2): 244-257.

Heinsch FA, Heilman JL, McInnes KJ, Cobos DR, Zuberer DA, Roelke DL (2004) Carbon dioxide exchange in a high marsh on the Texas Gulf Coast: effects of freshwater availability. Agricultural and Forest Meteorology 125(1-2): 159-172.

Jenkins JP, Richardson AD, Braswell BH, Ollinger SV, Hollinger DY, Smith M-L (2007) Refining light-use efficiency calculations for a deciduous forest canopy using simultaneous tower-based carbon flux and radiometric measurements. Agriculture and Forest Meteorology 143(1-2): 64-79.

Liu H, Randerson JT, Lindfors J, Chapin III FS (2005) Changes in the surface energy budget after fire in boreal ecosystems of interior Alaska: An annual perspective. Journal of Geophysical Research Atmospheres 110(D13).

Kato T, Tang Y, Gu S, Hirota M, Cui X, Du M, Li Y, Zhao X, Oikawa T (2004) Seasonal patterns of gross primary production and ecosystem respiration in an alpine meadow ecosystem on the Qinghai-Tibetan Plateau, China. Journal of Geophysical Research Atmospheres 109(D12).

Knohl A, Schulze ED, Kolle O, Buchmann N (2003) Large carbon uptake by an unmanaged 250-year-old deciduous forest in Central Germany. Agriculture and Forest Meteorology 118(3-4): 151-167.

Marcolla B, Cescatti A, Montagnani L, Manca G, Kerschbaumer G, Minerbi S (2005) Importance of advection in the atmospheric CO_2_ exchanges of an alpine forest. Agricultural and Forest Meteorology 130(193-206).

Marchesini LB, Papale D, Reichstein M, Vuichard N, Tchebakova N, Valentini R (2007) Carbon balance assessment of a natural steppe of southern Siberia by multiple constraint approach. Biogeosciences 4: 581-595.

Ma S, Baldocchi DD, Xu L, Hehn T (2007) Inter-annual variability in carbon dioxide exchange of an oak/grass savanna and open grassland in California. Agricultural and Forest Meteorology 147: 157-171.

McCaughey JH, Pejam MR, Arain MA, Cameron DA (2006) Carbon dioxide and energy fluxes from a boreal mixedwood forest ecosystem in Ontario, Canada. Agricultural and Forest Meteorology 140(1-4): 79-96.

Noormets A, Chen JQ, Crow TR (2007) Age-Dependent Changes in Ecosystem Carbon Fluxes in Managed Forests in Northern Wisconsin, USA. Ecosystems 10(2): 187-203.

Rey A, Pegoraro E, Tedeschi V, Parri I, Jarvis PG, Riccardo V (2002) Annual variation in soil respiration and its components in a coppice oak forest in central Italy. Global Change Biology 8(9): 851-866.

Rambal S, Joffre R, Ourcival JM, Cavender-Bares J, Rocheteau A (2004) The growth respiration component in eddy CO_2_ flux from a Quercus ilex mediterranean forest. Global Change Biology 10(9): 1460-1469.

Rannik U, Altimir N, Raittila J, Suni T, Gaman A, Hussein T, Holtta T, Lassila H, Latokartano M, Lauri A, Natsheh A, Petaja T, Sorjamaa R, Yla-Mella H, Keronen P, Berninger F, Vesala T, Hari P, Kulmala M (2002) Fluxes of carbon dioxide and water vapour over Scots pine forest and clearing. Agricultural and Forest Meteorology 111(3): 187-202.

Rinne J, Tuovinen JP, Laurila T, Hakola H, Aurela M, Hypen H (2000) Measurements of hydrocarbon fluxes by a gradient method above a northern boreal forest. Agricultural and Forest Meteorology 102(1): 25-37.

Scholes RJ, Gureja N, Giannecchinni M, Dovie D, Wilson B, Davidson N, Piggott K, McLoughlin C, Velde K, Freeman A, Bradley S, Smart R, Ndala S (2001) The environment and vegetation of the flux measurement site near Skukuza, Kruger National Park. Koedoe 44(1): 73-83.

Scott RL, Jenerette GD, Potts DL, Huxman TE (2009) Effects of seasonal drought on net carbon dioxide exchange from a woody-plant-encroached semiarid grassland. Journal of Geophysical Research: Biogeosciences 114(G4).

Tedeschi V, Rey A, Manca G, Valentini R, Jarvis PG, Borghetti M (2006) Soil respiration in a Mediterranean oak forest at different developmental stages after coppicing. Global Change Biology 12(1): 110-121.

Thomas CK, Law BE, Irvine J, Martin JG, Pettijohn JC, Davis KJ (2009) Seasonal hydrology explains inter-annual and seasonal variation in carbon and water exchange in a semi-arid mature Ponderosa Pine forest in Central Oregon. Journal of Geophysical Research: Biogeosciences 114(G4).

Urbanski S, Barford C, Wofsy S, Kucharik C, Pyle E, Budney J, McKain K, Fitzjarrald D, Czikowsky M, Munger JW (2007) Factors controlling CO_2_ exchange on timescales from hourly to decadal at Harvard Forest. Journal of Geophysical Research: Biogeosciences 112(G2).

Valentini R, De Angelis P, Matteucci G, Monaco R, Dore S, Scarascia-Mugnozza G (1996) Seasonal net carbon dioxide exchange of a beech forest with the atmosphere. Global Change Biology 2(3): 199–207.

van-Gorsel E, Leuning R, Cleugh HA, Keith H, Suni T (2007) Nocturnal carbon efflux: reconciliation of eddy covariance and chamber measurements using an alternative to the u*-threshold filtering technique, Tellus B 59(5), 397-403.

Vickers D, Thomas C, Law BE (2009) Random and systematic CO_2_ flux sampling errors for tower measurements over forests in the convective boundary layer. Agricultural and Forest Meteorology 149(1): 73-83.

Wirth C, Schulze ED, Kusznetova V, Milyukova I, Hardes G, Siry M, Schulze B, Vygodskaya NN (2002) Comparing the influence of site quality, stand age, fire and climate on aboveground tree production in Siberian Scots pine forests. Tree Physiology 22(8): 537-552.

Wallin G, Linder S, Lindroth A, Rantfors M, Flemberg S, Grelle A (2001) Carbon dioxide exchange in Norway spruce at the shoot, tree and ecosystem scale. Tree Physiology 21(12-13): 969-976.
